# Strong In-Plane Magnetization and Spin Polarization in (Co_0.15_Fe_0.85_)_5_GeTe_2_/Graphene van der Waals Heterostructure Spin-Valve at Room Temperature

**DOI:** 10.1021/acsnano.3c07462

**Published:** 2024-02-08

**Authors:** Roselle Ngaloy, Bing Zhao, Soheil Ershadrad, Rahul Gupta, Masoumeh Davoudiniya, Lakhan Bainsla, Lars Sjöström, Md. Anamul Hoque, Alexei Kalaboukhov, Peter Svedlindh, Biplab Sanyal, Saroj Prasad Dash

**Affiliations:** † Department of Microtechnology and Nanoscience, Chalmers University of Technology, SE-41296 Göteborg, Sweden; ‡ Department of Physics and Astronomy, Uppsala University, Box-516, 75120 Uppsala, Sweden; § Department of Materials Science and Engineering, Uppsala University, Box 35, SE-751 03 Uppsala, Sweden; ∥ Department of Physics, Indian Institute of Technology Ropar, Roopnagar 140001, Punjab, India; ⊥ Graphene Center, Chalmers University of Technology, SE-41296 Göteborg, Sweden

**Keywords:** van der Waals magnet, spin-valve, graphene, van der Waals heterostructures, 2D magnets, in-plane magnetization, spin polarization

## Abstract

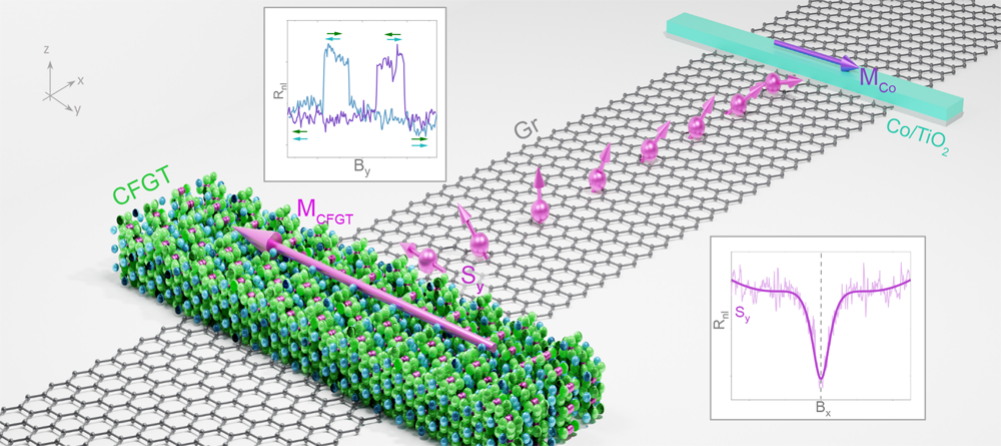

Van der Waals (vdW) magnets are promising, because of their tunable magnetic properties with doping or alloy composition, where the strength of magnetic interactions, their symmetry, and magnetic anisotropy can be tuned according to the desired application. However, so far, most of the vdW magnet-based spintronic devices have been limited to cryogenic temperatures with magnetic anisotropies favoring out-of-plane or canted orientation of the magnetization. Here, we report beyond room-temperature lateral spin-valve devices with strong in-plane magnetization and spin polarization of the vdW ferromagnet (Co_0.15_Fe_0.85_)_5_GeTe_2_ (CFGT) in heterostructures with graphene. Density functional theory (DFT) calculations show that the magnitude of the anisotropy depends on the Co concentration and is caused by the substitution of Co in the outermost Fe layer. Magnetization measurements reveal the above room-temperature ferromagnetism in CFGT and clear remanence at room temperature. Heterostructures consisting of CFGT nanolayers and graphene were used to experimentally realize basic building blocks for spin valve devices, such as efficient spin injection and detection. Further analysis of spin transport and Hanle spin precession measurements reveals a strong in-plane magnetization with negative spin polarization at the interface with graphene, which is supported by the calculated spin-polarized density of states of CFGT. The in-plane magnetization of CFGT at room temperature proves its usefulness in graphene lateral spin-valve devices, thus revealing its potential application in spintronic technologies.

Magnetism in van der Waals (vdW) materials offers an excellent platform for exploring fascinating spintronic and quantum science and technology.^1,2^ vdW magnetic materials are particularly interesting due to their tunable magnetic properties, where the magnetic anisotropy and Curie temperature (*T*_c_) can be tuned by alloying,^3,4^ doping,^5^ gating,^6,7^ proximity-induced coupling,^8^ pressure,^9^ and functionalization.^10^ The integration of vdW magnets in heterostructures with graphene, semiconductors, topological materials, and superconductors, can also result in interesting proximity-induced effects and strongly correlated electronic phenomena.^11^

By utilizing vdW magnets, several proof-of-concept spintronic and topological quantum phenomena have been demonstrated, such as spin valves,^12^ tunnel magnetoresistance,^13^ proximity magnetism,^14,15^ exchange bias,^16^ skyrmions,^17^ and spin–orbit torque.^18,19^ However, most of the demonstrated devices were limited by the low *T*_c_ of the vdW magnets, limiting their potential for applications. In the case of itinerant vdW ferromagnets, the family of Fe*_n_*GeTe_2_ is interesting for spintronic devices. After the discovery of Fe_3_GeTe_2_ with perpendicular magnetic anisotropy (PMA) and *T*_c_ around ∼200 K,^20^ Fe_5_GeTe_2_ has been shown to exhibit ferromagnetism up to nearly room temperature.^21,22^ Interestingly, canted PMA has also been reported in Fe_5_GeTe_2_/graphene spin-valve devices at room temperature.^23^ Despite these advances, spintronic devices using such high *T*_c_ vdW magnetic materials with in-plane magnetization are lacking so far. In-plane ferromagnets are specifically useful elements in unconventional neuromorphic computing,^24,25^ probabilistic computing,^26^ and spintronic memory devices,^27,28^ among others. Encouragingly, well beyond room-temperature magnetic order can be achieved in (Co_*x*_Fe_1–*x*_)_5_GeTe_2_,^4,29^ with the magnetic properties being tunable by the substitution of Fe with Co atoms.

Here, we demonstrate in-plane magnetization and spin polarization for beyond room-temperature vdW ferromagnet (Co_0.15_Fe_0.85_)_5_GeTe_2_ (CFGT). We fabricated vdW-based heterostructure spin-valve devices consisting of CFGT and chemical-vapor-deposited (CVD) graphene, which is a suitable channel material for long-distance spin transport and spin logic operations.^30,31^ Detailed spin-valve and Hanle spin precession measurements on CFGT/graphene hybrid devices demonstrate efficient spin injection, detection, transport, and precession functionalities. The observation of symmetric Hanle spin precession signals proves the in-plane magnetization and spin polarization of CFGT, whereas the polarity of the measured spin signals provides evidence of negative spin polarization at the CFGT/graphene interface. These findings are well-supported by density functional theory (DFT) calculations, showing composition-dependent modification of the magnetic anisotropy and spin-polarized density of states.

## Results/Discussion

**Figure 1:**
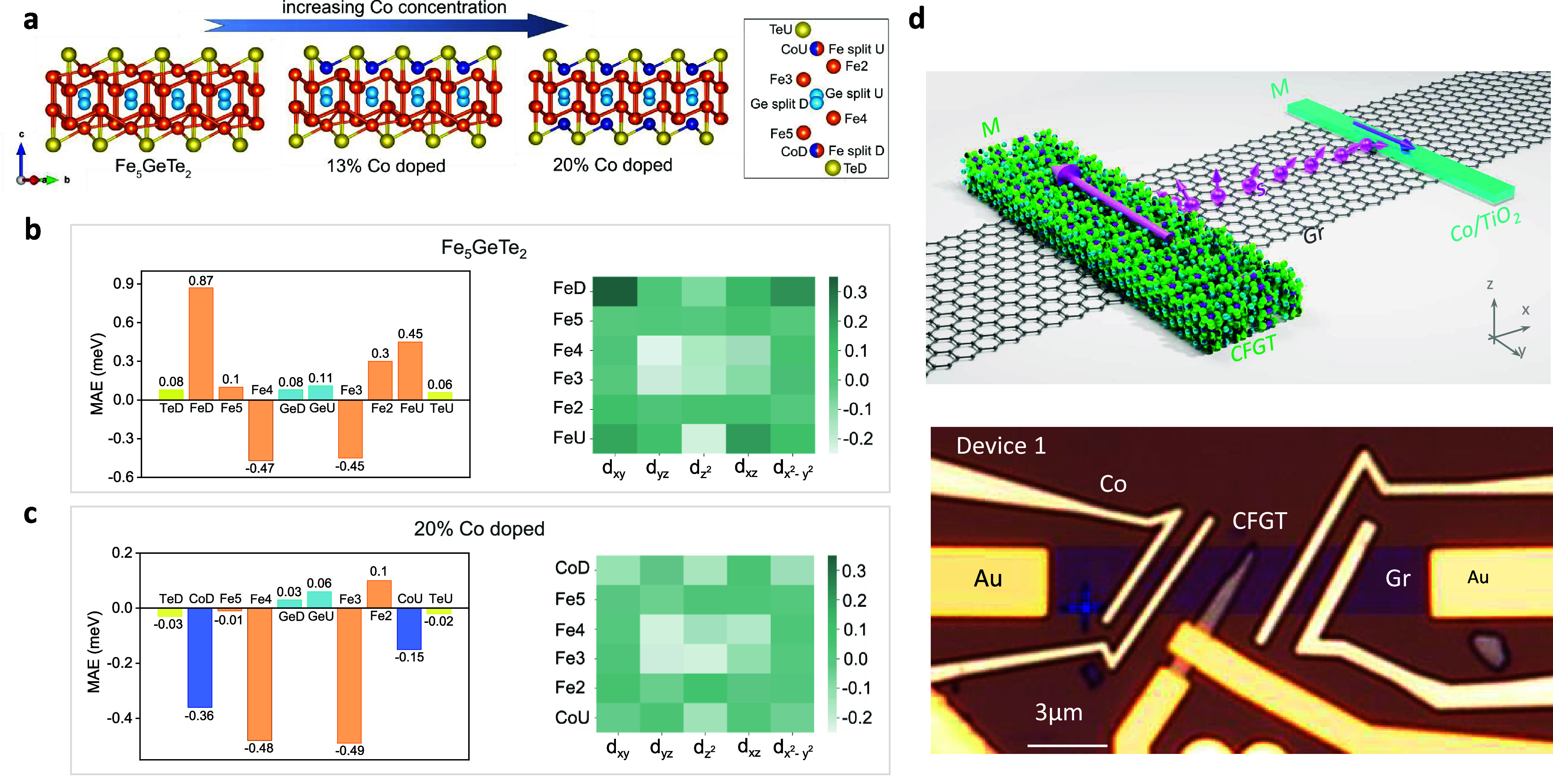
Composition-dependent magnetic anisotropy calculations of CFGT and CFGT/graphene vdW spin-valve device. (a) Schematic representation of CFGT structure as the concentration of Co atoms increases, occupying the outermost (split) sublattice of Fe, where dark blue, orange, yellow, and light blue atoms represent Co, Fe, Te, and Ge, respectively. The right panel shows atomic notations and their relative positions along the *c*-axis. (b) Atom-projected MAE in meV (left panel) and heat map of orbital projected MAE (right panel) resolved into 3d orbitals of Fe atoms for pure FGT. (c) Atom-projected MAE in meV (left panel) and heat map of orbital-projected MAE (right panel) for Fe and Co atoms in 20.1% doped CFGT. (d) Schematics and microscope picture of the CFGT/graphene lateral spin-valve device, where CFGT acts as a spin injector/detector and Co/TiO_2_ as a reference ferromagnetic contact on the CVD graphene channel. Nonmagnetic Ti/Au contacts are used for reference electrodes. Scale bar = 3 μm.

In the vdW ferromagnet CFGT, the magnetocrystalline anisotropy and Curie temperature (*T*_c_) are strongly dependent on Co doping, as schematically illustrated in Figure 1a. To understand the preferential doping site of Co atoms, we calculated the formation energy of Co in each sublattice (see details given in the Supporting Information and Table S1). Formation energies indicate that up to doping concentrations of 20.1%, Co atoms prefer to substitute to the outermost Fe sublattices, i.e., FeU and FeD species (dark blue color). This is compatible with previous results, showing that Fe split sites are most prone to defects.^21,32,33^ The magnetic anisotropy energy (MAE) of pristine and Co-doped structures in monolayer and bulk forms is listed in Table S2. Note that, in the absence of Co, the pure Fe_5_GeTe_2_ (FGT) monolayer has an out-of-plane easy magnetization axis, with MAE = +18.7 μeV/atom. With Co doping, the direction of the easy axis changes to in-plane. In the monolayer regime, the MAE values for Co concentrations of 6.7%, 13.4%, and 20.1% are −29.6, −79.1, and −149.2 μeV/atom, respectively. A similar trend is also observed in the bulk samples. A direct relation between the Co concentration and the MAE is evident, where doping can alter the magnetocrystalline anisotropy from a weak out-of-plane to a stronger in-plane magnetization.

To understand the origin of this magnetocrystalline anisotropy change, we have calculated the atom and orbital projected MAE for pure FGT and 20.1% CFGT, as shown in Figures 1b and 1c, respectively (for intermediate concentrations, see Figure S1 in the Supporting Information). In the case of pure FGT (Figure 1b), the main contribution to MAE comes from Fe atoms, while Ge and Te atoms have a marginal impact on the direction of the easy axis. Among the Fe sublattices, however, the contribution is not uniform. Outermost Fe atoms have large out-of-plane easy axis (0.87 and 0.45 meV per FeD and FeU, respectively). As we move toward the center of a monolayer, the size of out-of-plane magnetic anisotropy on Fe5 and Fe2 sublattices decreases to 0.10 for Fe5 and 0.30 meV/atom for Fe2. Notably, in the central region, Fe4 and Fe3 sublattices have large in-plane MAE of −0.47 and −0.45 meV/atom, respectively. Therefore, there is a competing interplay between the direction of magnetic anisotropy of various Fe sublattices that determines the overall easy axis of the pristine FGT crystal. In 20.1% doped CFGT, shown in Figure 1c, Co atoms (in dark blue bars), which have substituted Fe split site atoms, have a relatively large in-plane magnetic energy with −0.30 meV/atom for CoD and −0.15 meV/atom for CoU. Furthermore, we observe a decrease in the MAE values of the Fe5 and Fe2 sublattices, located next to Co dopants, with MAE values of −0.01 and 0.1 meV/atom, respectively. The central sublattices of Fe4 and Fe3 are marginally affected by Co atoms. Accordingly, one can understand that two congruent contributions add up to the change in the easy axis in the presence of Co atoms. First, and most importantly, is the substitution of largely out-of-plane magnetization in FeU and FeD with in-plane magnetization in CoU and CoD. Second, is the MAE reduction of 0.1 and 0.2 meV/atom on Fe5 and Fe2 atoms, respectively, which are located adjacent to Co dopants. The atom-projected MAEs for lower Co concentrations also show similar behaviors (see Figure S1), indicating that the physics behind the easy axis change acts independent of configuration or doping concentration.

We further provided the heat map of orbital-resolved MAE for d-orbitals of Fe and Co atoms in the right panel of Figures 1b and 1c, respectively. It was found that, in pure FGT (Figure 1b), the d_*xy*_ and d_*x*^2^–*y*^2^_ orbitals contribute strongly to the out-of-plane anisotropy of FeU and FeD atoms, while the in-plane tendency in Fe3 and Fe4 mainly comes from the d_*yz*_, d_*xz*_, and d_*z*^2^_ orbitals. In contrast, in the Co dopants (CoU and CoD) in CFGT (Figure 1c), the d_*xy*_ and d_*x*^2^–*y*^2^_ orbitals tend to have in-plane moments. Table S3 in the Supporting Information shows the average magnetic moments on different sublattices in FGT and 20.1% doped CFGT. One can see that Co atoms have a magnetic moment of 0.58 and 0.80 μ_B_, depending on their occupational site. This is significantly smaller than that of Fe split atoms (with 1.53 and 1.93 μ_B_ for FeU and FeD, respectively). Thus, Co atoms prefer to have a lower spin state, compared with Fe atoms in the same occupational site.

To experimentally verify the in-plane magnetization and spin polarization in CFGT, we fabricated lateral graphene spin-valve devices (Figure 1d) using CFGT grown by chemical vapor transport (CVT) method (from HQ Graphene). A lateral spin-valve is a basic building block of spintronic devices, where one can investigate several functionalities, such as spin injection, transport, precession and detection using vdW materials and hybrid structures. A schematic diagram and optical microscope picture of a lateral spin-valve device are illustrated in Figure 1d, where the spin current can be injected/detected by CFGT on a CVD graphene channel and detected/injected by a reference ferromagnetic Co/TiO_2_ contact (see the Methods section for fabrication details). As the aspect ratio of CFGT is an important factor in determining the magnetic shape anisotropy and the easy axis of magnetization, a very narrow CFGT flake (*W*_CFGT_ ≈ 0.7 μm and thickness of ∼30 nm) was used to achieve a stronger magnetic shape anisotropy and a nearly single magnetic domain state at the interface with graphene. The well-known in-plane magnetization and spin polarization of Co/TiO_2_ electrodes on our graphene channels should assist us in quantifying the magnetic properties of the vdW magnets.

**Figure 2:**
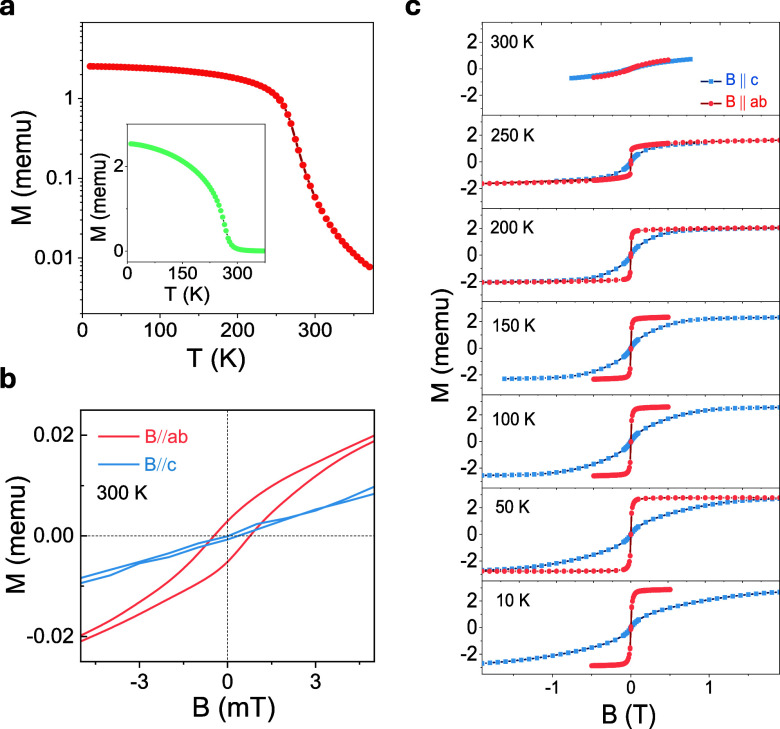
Beyond room temperature magnetism in CFGT. (a) Magnetic moment (*M*) on a logarithmic scale of bulk CFGT versus temperature for an applied magnetic field of 50 mT. The inset shows the *M*(*T*) behavior of CFGT using a linear scale for the magnetic moment. (b) Magnified magnetic hysteresis loops of CFGT with in-plane (orange) and out-of-plane (light blue) magnetic field sweeps at 300 K. (c) Magnetic hysteresis loops of the CFGT crystal with in-plane (orange) and out-of-plane (light blue) magnetic field sweeps in the temperature range of 10–300 K, showing strong in-plane magnetization maintained through the temperature range.

First, the temperature-dependent magnetic moment of a bulk CFGT crystal was measured with a superconducting quantum interference device (SQUID) magnetometer. Figure 2a shows that a higher *T*_C_ value (above 300 K) has been achieved. Magnetic hysteresis loops measured from 10 to 300 K for both in-plane (B//*ab*) and out-of-plane (B//*c*) orientations (Figures 2b and 2c) of the magnetic field show that strong in-plane magnetic magnetization is maintained up to room temperature. The magnified hysteresis loop at 300 K (Figure 2b) shows a clear remanence in the in-plane direction, whereas the magnetic hysteresis almost vanishes for the out-of-plane orientation. Such stabilization of in-plane magnetization agrees with the theoretical calculations presented above.

**Figure 3:**
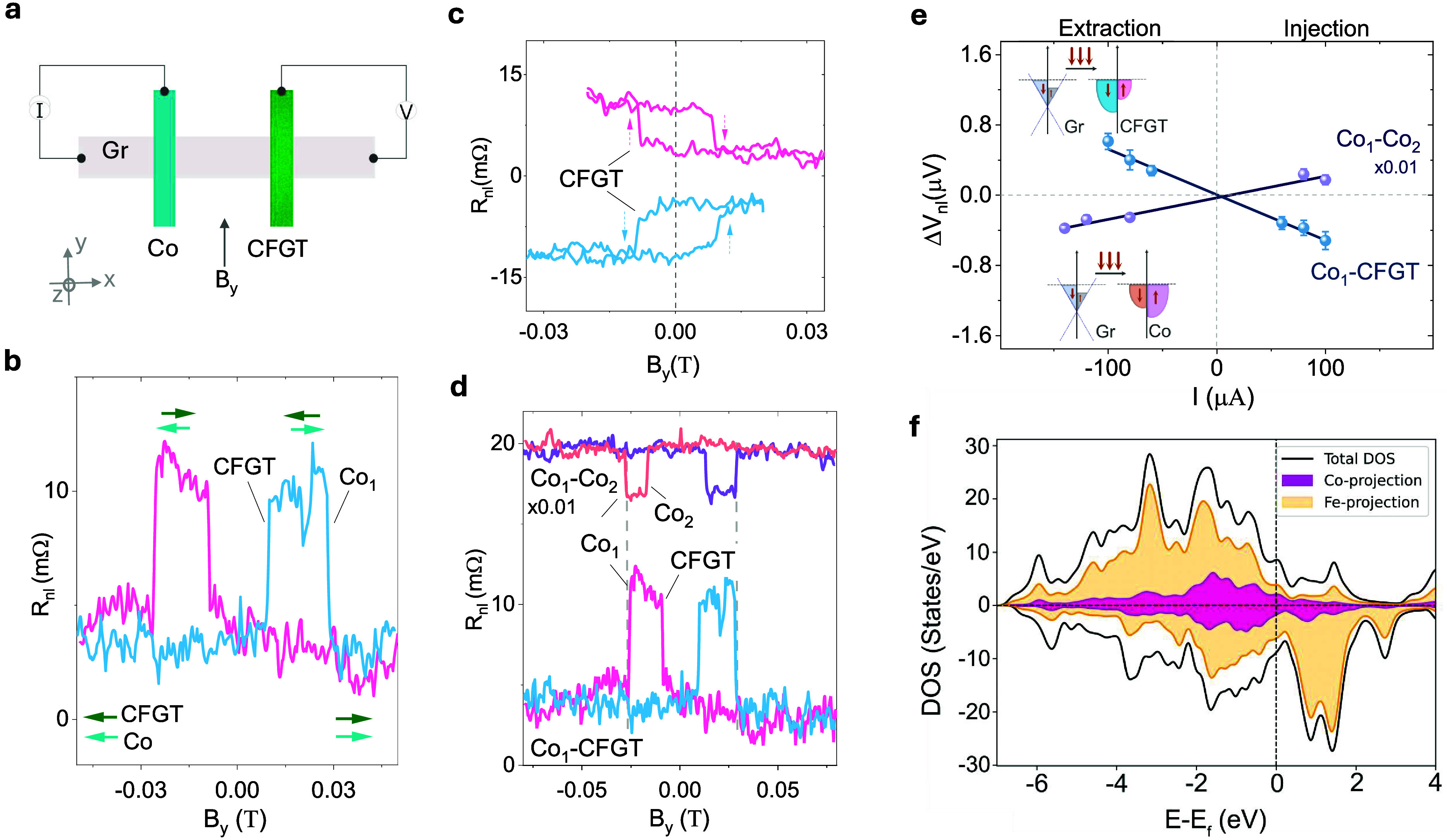
Lateral spin-valve with CFGT-graphene heterostructure at room temperature. (a) Schematic diagram for nonlocal spin-valve for Co(injection)-Gr-CFGT (detection) configuration for Dev 1. (b) Measured spin-valve signal for Dev 1, showing sharp switching for both CFGT and Co magnetic contacts. The arrows represent the direction of magnetization of CFGT and Co through a magnetic sweep. (c) Minor-loop measurements of CFGT. The arrows indicate the CFGT magnetic moment switching under the up- and down-sweep of the magnetic field. (d) Comparison of measured nonlocal spin-valve signals of Co-Gr-CFGT and Co-Gr-Co devices, showing the opposite sign for the same polarity of applied bias current of 100 μA. The signals are shifted along the *y*-axis, and the signal for Co-Gr-Co was rescaled by ×0.01 for clarity. (e) Bias dependence of the spin signal for both Co-Gr-CFGT and Co-Gr-Co spin-valve devices in the spin injection and extraction regimes, showing opposite spin polarization for Co and CFGT contacts. The error bars are estimated from the standard deviation of the measured data. The insets show the detection mechanism of the spin current for Co_1_–CFGT and Co_1_–Co_2_ devices. (f) DOS (solid black line) and pDOS (colored shaded) for 20.1% doped CFGT, where orange represents the density of Fe states and magenta represents the density of Co states.

The lateral CFGT/graphene heterostructure spin-valve device can probe the interfacial spin polarization and, hence, the magnetic anisotropy of thin CFGT flakes. We performed nonlocal spin transport measurements on a graphene spin valve to quantify the injected (detected) spin current by a thin CFGT flake (∼30 nm). Figure 3a illustrates the nonlocal measurement configuration, where the current is passed through the Co-graphene interface (injector circuit), and voltage is measured at the CFGT–graphene interface (detector circuit). Figure 3b shows the nonlocal spin-valve data for the detection of spin current by CFGT (Dev 1) with the magnetic field sweep along the *y*-axis (B_*y*_) at room temperature. This allows the control of the relative orientation of the magnetic moment of the injector (Co) and detector (CFGT) from parallel to antiparallel orientations resulting in the spin-valve signal with two resistance states. Since the reference Co contacts have a magnetic easy axis along the *y*-direction, the observation of a spin-valve signal confirms the detection of in-plane S_*y*_ spins by the detector CFGT contact. Noticeably, the CFGT shows sharp switching with a clear remanence and hysteresis in the spin-valve signals, indicating the presence of dominant S_*y*_ spin polarization. This is also replicated in minor loop measurements with forward and backward field sweeps before reaching the Co coercive field. The data measured in the two magnetization configurations show an apparent memory effect (Figure 3c). Spin detection by CFGT using a different contact in Dev 1 is presented in Figure S2, and spin injection by CFGT is also measured in another device (Dev 2), as presented in Figure S3 in the Supporting Information.

We performed control measurements to probe the sign of spin polarization at the CFGT/graphene interface and compared it to the standard Co/graphene contacts by measuring a purely Co–Co (injector-detector) device. It is well-established that Co has a positive spin polarization, which means that the majority of spins at the Fermi level are parallel to its bulk magnetization.^34^ However, we observed a reversal of the spin-valve signal when we compared Co-CFGT to Co–Co at the same bias condition (Figure 3d). We plot the detailed bias-dependent spin signal for Dev 1 in Figure 3e, where the polarity of the spin-valve signal for the Co-CFGT and Co–Co devices changes according to the polarity of the bias current and follows a linear trend for the range of current considered. Note that the opposite polarity for the measured spin-valve signals for Co-CFGT and Co–Co remains consistent throughout the bias range considered. For different current bias polarities, the mechanism for spin accumulation at the interface of the injector magnet and the graphene transport channel changes between spin injection (+I_dc_) or spin extraction (−I_dc_). Considering the Co injector, for positive bias, spin-polarized electrons tunnel from Co into the graphene channel, accumulating a spin population at the interface with polarization in accordance with the spin polarization of Co. On the other hand, when we apply a negative bias, electrons from graphene tunnel into the injector. In this case, since there are more available states for majority spin electrons in Co, more majority spin electrons will be extracted from the graphene channel, creating a nonequilibrium spin population in graphene dominated by minority spin electrons, as illustrated in the inset of Figure 3e. This can further be analyzed by looking at the expression for the amplitude of the nonlocal spin-valve signal given below:^35^
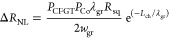
1where *P*_CFGT_ and *P*_Co_ are the spin polarization of CFGT and Co, respectively; *λ*_gr_ is the spin diffusion length in graphene; *R*_sq_ is the graphene square resistance; and *L*_ch_ and *w*_gr_ are the graphene channel length and width, respectively.

The opposite nonlocal spin-valve signals observed in these devices indicate opposite spin polarizations of the Co and CFGT contacts on graphene. This means that CFGT has a negative spin polarization and that the minority density of states is larger than the majority density of states at the Fermi level. In this case, when we apply a bias on the Co injector, and align the magnetic moments of Co and CFGT, the spin polarizations of the detector and injector have opposite directions, hence opposite signs are expected for the Co–Co and Co-CFGT devices, in accordance with eq 1. This observation is further substantiated by our calculations of the density of states (DOS) and the projected density of states (pDOS) for CFGT. Figure 3f and Figure S4 show the total DOS and pDOS for 20.1% and 13.4% doped CFGT, respectively. The spin polarization can be calculated by using the expression
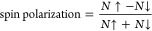
where *N* is the density of states at the Fermi level (solid vertical black line), and the arrows ↑ and ↓ correspond to the majority and minority spin directions, respectively. Accordingly, a negative spin polarization of −2.1% and −7.9% was calculated for 20.1% and 13.4% CFGT, respectively. These are smaller than that of pure FGT with −10% spin polarization.^23^ The accumulated pDOS of the Fe atoms, shown by orange shading in Figure 3, has the largest contribution to the total DOS and, thus, the two curves resemble each other significantly. However, right at the Fermi level, the Co atoms have a positive spin polarization (violet-shaded curve), which competes with the overall negative polarization of the Fe atoms, decreasing the magnitude of the negative spin polarization. Consequently, the magnitude of spin polarization should decrease as the Co atom concentration increases, hence CFGT has a smaller spin polarization, compared to pure FGT. Atom-resolved spin polarizations are listed in Table S3 in the Supporting Information. Here, the main compensation comes from CoU with +17.5% spin polarization and substituting FeU with −31.6% spin polarization.

**Figure 4:**
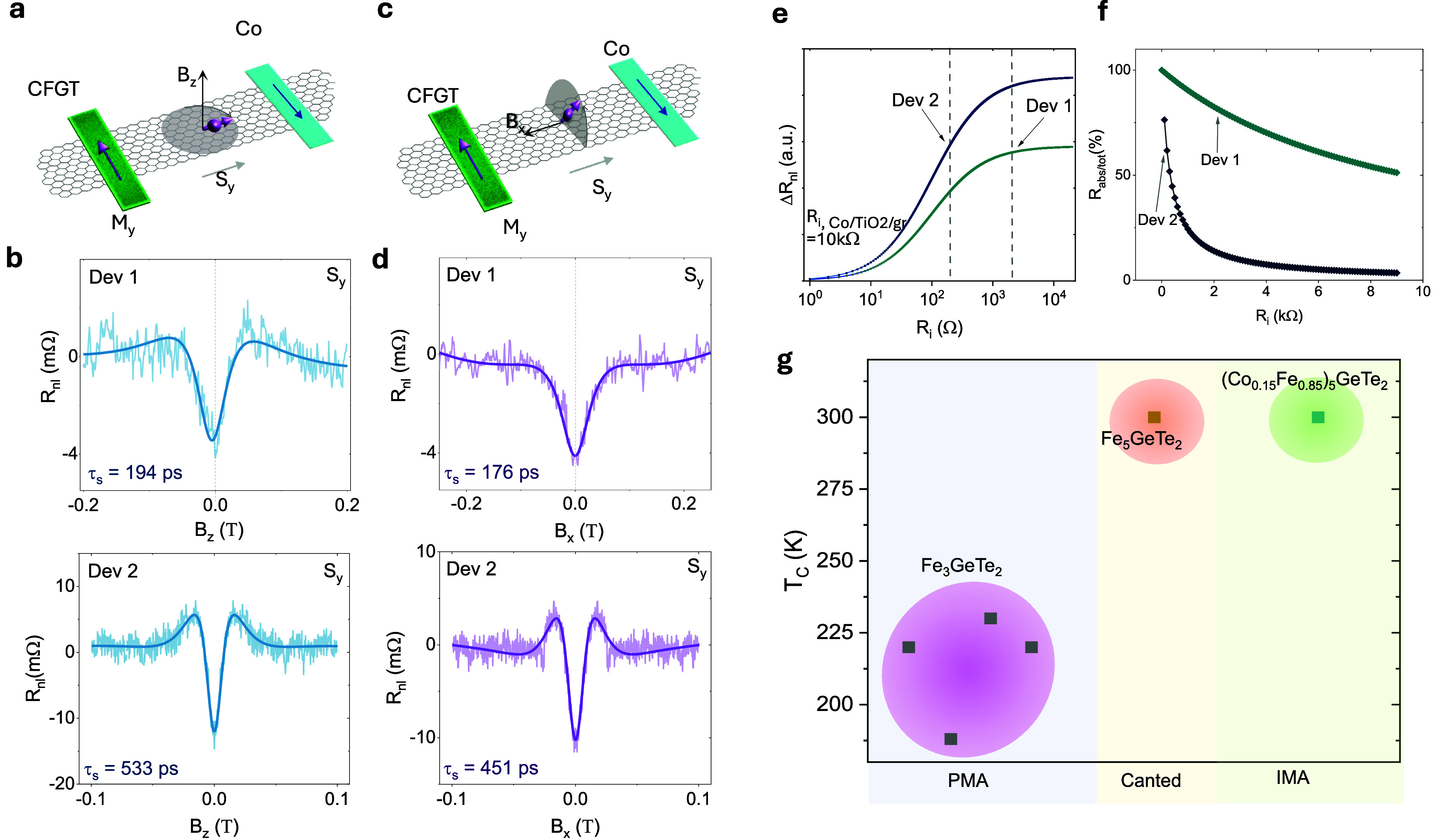
Hanle spin precession measurements in the CFGT–graphene heterostructure at room temperature. (a) Schematic diagram of the z-Hanle spin precession measurement in the CFGT-graphene spin-valve. The magnetic field is applied along the *z*-direction, and M_*y*_ is the magnetic moment of CFGT along the *y*-axis. (b) Measured symmetric *z*-Hanle spin precession signal and the data fitting for Dev 1 (CFGT as detector) and Dev 2 (CFGT as injector), showing the dominant S_*y*_ spin polarization component of CFGT at room temperature. A linear background is subtracted from the measured data. (c) Schematic diagram of Hanle spin precession measurement showing applied magnetic field along the *x*-direction. Similarly, M_*y*_ is the magnetic moment of CFGT along the *y*-axis. (d) The measured symmetric *x*-Hanle spin precession signal and data fitting for Dev 1 and Dev 2 show the dominant S_*y*_ spin polarization component of CFGT at room temperature. A linear background is subtracted from the measured data. (e) Calculated nonlocal spin signal as a function of the CFGT-graphene interface resistance *R*_i_ in the CFGT/Gr/Co lateral spin-valve device. Dashed lines indicate the measured CFGT-Gr interface resistances in our devices. (f) Calculated spin absorption ratio as a function of the interface resistance *R*_i_ and square resistance *R*_sq_ of the graphene channel. (g) Comparison of our results to reported magnetic tunnel junctions and spin-valve devices based on the family of vdW ferromagnets Fe_3_GeTe_2_ and Fe_5_GeTe_2_.^12,13,23,36,37^

To unambiguously prove the spin signal in our devices, we conducted Hanle spin precession experiments^38^ in both the *z*-axis (z-Hanle) and the *x*-axis (x-Hanle) geometries. This helps to evaluate the spin lifetime and diffusion length in the graphene channel and to estimate the spin polarization of the CFGT/graphene interface in different orientations, which provides a direct probe to the direction of the magnetic moment of CFGT. In *z*-Hanle, the out-of-plane magnetic field B_*z*_ drives the injected spins to precess in the *x*–*y* plane, as illustrated in Figure 4a. Spin precession results in a B_*z*_-dependent evolution of the nonlocal spin signal. The measured nonlocal Hanle signal is proportional to the spin polarization of injector and detector (*R*_nl_∝ *P*_in_·*P*_de_), with P_in(de)_ being the spin polarization of the injector (detector), and a symmetric (antisymmetric) signal corresponds to an initial S_*y*(*x*)_ spin state. As presented in Figure 4b, for Dev 1, the data for the nonlocal *z*-Hanle signal constitutes a symmetric curve suggesting a parallel state of the injected spins S_*y*_ with the magnetic moments of the CFGT detector. For Dev 2, the signal is obtained by presetting the injector (CFGT) and detector (Co) to parallel and antiparallel states (the raw data and analysis are shown in Figure S5). The decomposed average *z*-Hanle signal shows only symmetric components, which means that, like Dev 1, the injected spins by CFGT have only components along the *y*-axis, S_*y*_. Similarly, the *x*-Hanle measurement is performed with the external B_*x*_ field applied along the *x*-axis, inducing a spin precession in the *yz*-plane (Figure 4c). The observation of a symmetric *x*-Hanle curve in Figure 4d, for Dev 1 and Dev 2, demonstrates the dominant in-plane spin component S_*y*_ at the CFGT/graphene interface. From these precession measurements, we excluded any out-of-plane (S_*z*_) contributions for both Dev 1 and Dev 2, proving the strong in-plane magnetization of CFGT. In addition, by choosing flakes with the long axis along the *y*-direction, we were able to obtain only S_*y*_ components, which translates to a magnetic moment in CFGT that is along the *y*-direction (M_*y*_). This is different from earlier reported Hanle precession measurements in Fe_5_GeTe_2_/graphene spin valve, where contributions from S_*x*_, S_*y*_, and S_*z*_ were observed pointing to a canted perpendicular magnetization of Fe_5_GeTe_2_.^23^

By fitting the measured signal using the Hanle formula (eq 2),^35^ we evaluate the spin lifetime and the spin diffusion length in graphene channel from *z*-Hanle to be τ_s_ = 194 ± 29 ps and λ_gr_ = 2.1 ± 0.3 μm for Dev 1; and τ_s_ = 533 ± 20 ps and λ_gr_ = 4.27 ± 0.2 μm for Dev 2. From *x*-Hanle, we extract comparable spin transport parameters: τ_s_ = 176 ± 14 ps and λ_s_ = 2.5 ± 0.2 μm for Dev 1; and τ_s_ = 451 ± 18 ps and λ_I_ = 3.42 ± 0.1 μm for Dev 2.
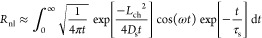
2Here, *L*_ch_ is the graphene channel length, *D*_s_ is the spin diffusion constant, *τ*_s_ is the spin lifetime, and ω is the Larmor precession frequency.

We can extract the spin polarization at the CFGT/Gr interface (for Dev 2) to be 4.93% and 4.5%, using eqs 1 and 2, respectively, considering a measured spin polarization of 4.8% in the reference Co/TiO_2_/Gr contact (spin transport in the Co–Co reference for Dev 2 is presented in Figure S6 in the Supporting Information). The spin injection efficiency can be influenced by the conductance mismatch and spin absorption effects between the ferromagnet and graphene. Since the resistance in graphene and ferromagnets differ by a few orders of magnitude, usually a tunnel barrier is introduced to improve conductance matching.^39^ To assess the impact of the conductance mismatch between CFGT and graphene, we look at the effect of the CFGT/Gr interface resistance on the nonlocal spin-valve signal by employing the drift-diffusion model.^40^ For our calculations shown in Figure 4e, we set the Co/TiO_2_/Gr interface resistance to 10 kΩ, which is in the same range as the values measured for Dev 1 and Dev 2. The difference between calculated and nominal values for the two devices stems from the different *R*_sq_ of the graphene channel (Figure S7). For Dev 1, the CFGT/Gr interface resistance (*R*_*i*_) is 2 kΩ (with *R*_*i*_ · Area = 4.2 kΩ μm^2^), which is sufficient to obtain near-optimal nonlocal signal for this specific set of parameters. For Dev 2, with a more transparent contact of 200 Ω (with *R_i_* · Area = 1.3 kΩ μm^2^), the calculated signal obtained is lower than the nominal nonlocal signal, reflecting a more pronounced effect of conductance mismatch and spin-absorption effects in Dev 2. With optimal conductance matching, which can be obtained by incorporating a tunnel barrier, the projected spin polarization could reach up to ∼6.57%, which is in good agreement with the theoretical calculations discussed above. These analyses show the effect of interface resistance variations in the CFGT/Gr heterostructure on the nonlocal spin signal and spin injection efficiency. Further optimization of the interface resistance by using appropriate 2D tunnel barriers can provide higher spin injection efficiency into graphene.

To further evaluate how the effect of CFGT/Gr interface resistance on spin absorption in the graphene transport channel, we calculate the spin absorption rate (Γ) using an approximate model:^41^
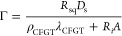
3where ρ_CFGT_ and λ_CFGT_ are the spin resistivity and spin diffusion length in CFGT, respectively. *R*_*i*_ is the interface resistance of the CFGT/Gr heterostructure and *A* refers to the interfacial area. Due to CFGT’s magnetic nature, ρ_CFGT_ · λ_CFGT_ becomes significantly less^42^ than *R*_*i*_ · *A*, resulting in a spin absorption rate that is dominated by the graphene resistance, spin relaxation in graphene, and interface resistance. Furthermore, the total spin relaxation time can be expressed as τ_s,total_ = τ_soc_ + τ_abs_, where τ_abs_ = 1/Γ and τ_SOC_, spin lifetime from SOC, was extracted from the spin parameters obtained in the experiment.^43^ We obtained a spin absorption contribution of 80% for Dev 1 and 61% for Dev 2, as illustrated in Figure 4f. The higher spin absorption for Dev 1 is due, in part, to the high graphene channel resistance of ∼3.3 kΩ, which is one order of magnitude higher, compared to Dev 2 (Figure S7). By increasing the interface resistance, which could be done by integrating a tunnel barrier, we can reduce the spin absorption contribution.

Lastly, to rule out any contribution from spin-related thermal effects due to a current bias, we evaluate how thermal spin injection from the ferromagnet^44^ and thermoelectric spin voltage in graphene^45^ affected our measured spin signal. First, we consider thermal spin injection from the ferromagnet, which can contribute to an additional spin current *J*_thermal ↑,↓_ ≈ *S*_↑,↓_ · ∇*T*. Note that the resulting thermal spin current depends on the thermal gradient ∇*T*, which scales with *I*^2^, moderated by the spin-dependent Seebeck coefficient. However, from bias dependent measurements performed on Co-CFGT Dev 1 shown in Figure 3e, the nonlocal spin valve signal varies linearly with the bias current. Moreover, there is no significant asymmetry of the magnitude of the spin signal for majority (spin injection) and minority (spin extraction) accumulation regimes. These indicate that thermal spin injection from the ferromagnet is negligible up to ±100 μA. Second, we look into the possible thermal contribution by thermoelectric spin voltage (TSV) in graphene. This effect has an enhanced contribution on the nonlocal spin signal at the charge neutrality point and decreases with charge carrier doping. We expect the contribution from TSV to be minimal since Dev 1 and Dev 2 are both measured away from the charge neutrality point, as shown in Figure S7.

From both theoretical and experimental findings, we can conclude the presence of a strong in-plane magnetization in CFGT, modulated by shape anisotropy, resulting in S_*y*_ spin polarization at the CFGT/graphene interface at room temperature. In comparison to earlier reports for the family of FGT vdW magnets (Figure 4g), our results highlight the in-plane magnetization and high *T*_c_ of CFGT in contrast to the out-of-plane magnetization in Fe_3_GeTe_2_ and canted magnetization in Fe_5_GeTe_2_.

## Conclusions

In summary, our findings reveal strong in-plane magnetization and spin polarization in the vdW magnetic metal CFGT at room temperature. Based on DFT calculations, this stems from the substitution of the outermost Fe atoms, having an out-of-plane easy axis, with Co dopants having an in-plane tendency. We demonstrated the utilization of such a high *T*_c_ vdW magnet in CFGT/graphene heterostructure spin-valve devices at room temperature. The in-plane magnetization and spin polarization in CFGT were probed by using spin-valve and Hanle spin precession measurement geometries, which provide insights into the room-temperature magnetism and spin polarization at the CFGT–graphene heterostructure interface. Spin injection, detection, and transport have been observed with a negative spin polarization of ∼5% at the CFGT/graphene interface. The symmetric Hanle curves measured in the devices prove the in-plane spin polarization of CFGT at room temperature, which was further verified through DFT calculations. These results have established the integration of an in-plane vdW ferromagnet with graphene in spin-valve devices, which is a very good indication of its considerable potential in the development of room-temperature 2D spintronic applications.

## Methods/Experimental

### DFT Calculations

Structural optimization and formation energy calculations were done by the Vienna Ab initio Simulation Package (VASP).^46,47^ The exchange-correlation potential was approximated by the generalized gradient approximation (GGA) with the Perdew, Burke, and Ernzerhof (PBE) functional.^48^ For integration in the Brillouin zone, we used a 11 × 11 × 1 and 11 × 11 × 3 *k*-point grid in the Monkhorst–Pack scheme^49^ for a √3 × √3 supercell of monolayer and bulk CFGT, respectively. The equilibrium lattice constants and atomic positions were optimized through energy minimization by using the conjugate gradient method up to the point that the force components on each atom were below 0.01 eV/Å. In the monolayer regime, the interaction between periodic images along the *z*-axis was minimized by adding a vacuum spacing of at least 20 Å. In all calculations, vdW correction via the DFT-D3 method of Grimme with zero-damping function was enabled. The electronic and magnetic properties were calculated by the QuantumATK-Synopsys package version Q-2021,^50^ using LCAO basis set, and “PseudoDojo” presudopotential.^51^ A density mesh cutoff of 140 hartree and *k*-point grids of 15 × 15 × 1 and 15 × 15 × 3 were used for monolayer and bulk self-consistent calculations. The magnetic anisotropy energy was calculated based on the force theorem, with *k*-point grids of 25 × 25 × 1 and 25 × 25 × 3 for monolayer and bulk, respectively, using the expression
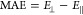
where *E*_⊥_ and *E*_∥_ denote out-of-plane and in-plane total magnetic energies, respectively.

### Fabrication of Devices and Electrical Measurements

The (Co_0.15_Fe_0.85_)_5_ GeTe_2_ (CFGT) nanolayer flakes (with a thickness of 20–30 nm), were exfoliated and dry-transferred onto a CVD graphene channel on an *n*^++^Si/SiO_2_ (285 nm) substrate inside a N_2_ glovebox. CVD graphene channels were prepared by electron beam lithography (EBL) and oxygen plasma patterning. For the fabrication of spin valve devices, nonmagnetic (Au/Ti) and magnetic contacts (Co/TiO_2_) were prepared using multiple EBL processes and electron beam evaporation of metals. The Au/Ti contacts were first evaporated on CFGT flakes after a few seconds of Ar ion milling to clean the surface. After which, another round of EBL and Au/Ti evaporation was performed for reference electrodes in graphene. Lastly, the ferromagnetic contacts of Co (60 nm)/TiO_2_(∼1–2 nm) on graphene were prepared using a two-step deposition process. Specifically, 0.4 nm of Ti was deposited two times, followed by a 10 Torr O_2_ oxidation for 10 min each, and then followed by 60 nm of Co deposition. The magnetic Co/TiO_2_ contacts were designed with varying widths (400–500 nm) to serve as a reference spin injector (detector), taking advantage of the well-defined magnetic properties of Co with in-plane magnetization controlled by strong shape anisotropy. The devices were not capped to preserve the graphene transport channel. The channel length and width of graphene in Dev 1 were ∼4.5 μm and ∼3 μm, respectively. The CFGT/Gr interface resistance was in the range of 1–3 kΩ, while the resistance of Co/TiO_2_/Gr contacts was ∼10–20 kΩ. For Dev 2, the graphene channel length and width were ∼13.7 μm and ∼3 μm, respectively. The CFGT/Gr interface resistance ranged from ∼150 to 200 Ω, while the resistance of Co/TiO_2_/Gr contacts were ∼7–25 kΩ.

The measurements were carried out at room temperature under vacuum conditions using a magnetic field sweep and a sample rotation stage. The electronic measurement system is composed of a current source (Keithley, Model 6221), a nanometer (Keithley, Model 2182A), and a dual-channel source meter (Keithley, Model 2612B).

### SQUID Measurements

A Quantum Design superconducting quantum interference device (SQUID) magnetometer was used to measure the static magnetic properties of bulk CFGT crystals. The bulk crystal was attached to a Si substrate to properly align the magnetic field during hysteresis measurements in both in-plane (B//*ab*) and out-of-plane (B//*c*) configurations.

## Data Availability

The data that support the findings of this study are available from the corresponding authors on a reasonable request.

## Supporting Information Available

Additional information and figures regarding structural and magnetic analysis from first-principle calculations (formation energy of Co substitution in different Fe sites; magnetic anisotropy energy for intermediate Co-doped FGT and for monolayer vs bulk case; and atom projected spin polarization for pristine FGT vs 20% Co-doped FGT); nonlocal spin-valve measurements in Dev 1; spin-valve signal with CFGT as injector and Co as detector for Dev 2; density of states (DOS) and projected density of states (pDOS) for 13.4% doped CFGT; detailed x-Hanle and z-Hanle measurements for Dev 2; spin transport in Co–Co (injector-detector) reference for Dev 2; square resistance of the graphene channel for Dev 1 and Dev 2 (PDF)
