# Supplementary material for: Strong In-Plane Magnetization and Spin Polarization in (Co0.15Fe0.85)5GeTe2/Graphene van der Waals Heterostructure Spin-Valve at Room Temperature
Source: ACS Nano. 2024 Feb 8;18(7):5240–8. doi: 10.1021/acsnano.3c07462 (PMC10883121; doi:10.1021/acsnano.3c07462)
Supplement: Supplementary file 1 — nn3c07462_si_001.pdf [file nn3c07462_si_001.pdf]

Supplementary Information

Strong in-plane magnetization and spin polarization in $(Co_{0.15}Fe_{0.85})_5GeTe_2$/graphene van der Waals heterostructure spin-valve at room temperature

Roselle Ngaloy[1], Bing Zhao[1], Soheil Ershadrad[2], Rahul Gupta[2,3], Masoumeh Davoudiniya[2], Lakhan Bainsla[4,1], Lars Sjöström[1], Md. Anamul Hoque[1], Alexei Kalaboukhov[1], Peter Svedlindh[3], Biplab Sanyal[2], Saroj Prasad Dash[1,5*]

[1]Department of Microtechnology and Nanoscience, Chalmers University of Technology, SE-41296, Göteborg, Sweden
[2]Department of Physics and Astronomy, Uppsala University, Box-516, 75120 Uppsala, Sweden
[3]Department of Materials Science and Engineering, Uppsala University, Box 35, SE-751 03 Uppsala, Sweden
[4]Department of Physics, Indian Institute of Technology Ropar, Roopnagar 140001, Punjab, India
[5]Graphene Center, Chalmers University of Technology, SE-41296, Göteborg, Sweden

Methods

DFT Calculations: Structural optimization and formation energy calculations were done by the Vienna Ab initio Simulation Package (VASP).[1,2] The exchange-correlation potential was approximated by the generalized gradient approximation (GGA) with the Perdew, Burke, and Ernzerhof (PBE) functional.[3] For integration in the Brillouin zone, we used a 11×11×1 and 11×11×3 k-point grid in the Monkhorst-Pack scheme[4] for a $\sqrt{3} \times \sqrt{3}$ supercell of monolayer and bulk CFGT, respectively. The equilibrium lattice constants and atomic positions were optimized through energy minimization, using the conjugate gradient method up to the point that the force components on each atom were below 0.01 eV/Å. In the monolayer regime, the interaction between periodic images along the z-axis was minimized by adding a vacuum spacing of at least 20 Å. In all calculations, vdW correction via DFT-D3 method of Grimme with zero-damping function was enabled. The electronic and magnetic properties were calculated by the QuantumATK-Synopsys package version Q-2021,[5] using LCAO basis set, and "PseudoDojo" presudopotential.[6] A density mesh cut-off of 140 Hartree and a k-point grid of 15×15×1 and 15×15×3 were used for monolayer and bulk self-consistent calculations. The magnetic anisotropy energy was calculated based on the force theorem, with a k-point grid of 25×25×1 and 25×25×3 for monolayer and bulk, respectively, using the expression: $MAE = \mathrm{E}\perp - \mathrm{E}\parallel$, where E⊥ and E∥ denote out-of-plane and in-plane total magnetic energies, respectively.

Fabrication of devices and electrical measurements: The $(Co_{0.15}Fe_{0.85})_5GeTe_2$ (CFGT) nanolayer flakes (with a thickness of 20-30 nm), were exfoliated and dry-transferred onto a CVD graphene channel on an $n^{++}Si/SiO_2$ (285 nm) substrate inside a N2 glovebox. CVD graphene channels were prepared by electron beam lithography (EBL) and oxygen plasma patterning. For the fabrication of spin valve devices, non-magnetic (Au/Ti) and magnetic contacts (Co/TiO_2) were prepared using multiple EBL processes and electron beam evaporation of metals. The Au/Ti contacts were first evaporated on CFGT flakes after a few seconds of Ar ion milling to clean the surface. After which, another round of EBL and Au/Ti evaporation was performed for reference electrodes in graphene. Lastly, the ferromagnetic contacts of Co (60 nm)/TiO_2(~1-2 nm) on graphene were prepared using a two-step deposition process. Specifically, 0.4 nm of Ti was deposited two times, followed by a 10 Torr O_2 oxidation for 10 minutes each, and then followed by 60 nm of Co deposition. The magnetic Co/TiO_2 contacts were designed with varying widths (400 - 500 nm) to serve as reference spin injector (detector), taking advantage of the well-defined magnetic properties of Co with in-plane magnetization controlled by strong shape anisotropy. The devices were not capped to

preserve the graphene transport channel. The channel length and width of graphene in Dev 1 were ~ 4.5 µm and ~ 3 µm, respectively. The CFGT/Gr interface resistance was in the range of 1-3 kΩ, while the resistance of Co/TiO_2/Gr contacts were around 10-20 kΩ. For Dev 2, the graphene channel length and width were ~13.7 µm and ~3 µm, respectively. The CFGT/Gr interface resistance ranged from ~150 Ω to 200 Ω, while the resistance of Co/TiO_2/Gr contacts were around 7-25 kΩ.

The measurements were carried out at room temperature under vacuum conditions using magnetic field sweep and a sample rotation stage. The electronic measurement system is composed of a current source (Keithley 6221), a nanometer (Keithley 2182A), and a dual-channel source meter (Keithley 2612B).

SQUID Measurements: A Quantum Design superconducting quantum interference device (SQUID) magnetometer was used to measure the static magnetic properties of bulk CFGT crystals. Both in-plane (B∥xy) and out-of-plane (B∥z) magnetic hysteresis loops were measured. The bulk CFGT crystal was glued on a Si substrate to properly align the magnetic field during the measurements.

Supplementary Note 1. Supplementary structural and magnetic analysis from first principles

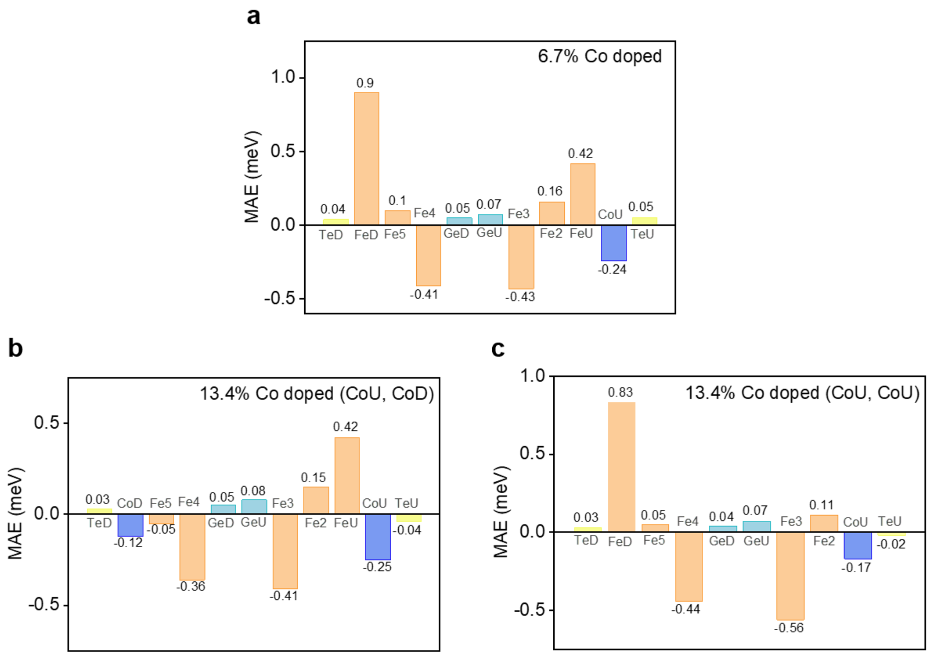

Supplementary Fig. S1. Atom projected MAE (in units of meV) *(a) for 6.7%, (b) 13.4% CFGT (CoU CoD configuration) and (c) 13.4% CFGT (CoU CoU configuration).*

Fe_5GeTe_2 (FGT) has 5 distinct Fe sublattices, where the outermost sublattice splits to partially occupy the uppermost (denoted by FeU) and lowermost (denoted by FeD) Fe layers. The rest of Fe sublattices are located between FeU and FeD, denoted by Fe2 - Fe5. To correspond to the splitting of FeU and FeD, Ge atoms also occupy split positions denoted by GeD and GeU. To be able to account for these split sites, we created a $\sqrt{3}\times\sqrt{3}R30°$ superstructure of FGT with 15 Fe atoms in the monolayer and 45 Fe atoms in ABC stacked bulk. Accordingly, we distributed split site Fe atoms into two FeU (Fe rich) and one FeD (Fe deficient). In an earlier study, we found that this special superstructure configuration is energetically favored by 0.1 eV/f.u. over the primitive unit cell of FGT[7]. Moreover, there are traces of $\sqrt{3}\times\sqrt{3}R30°$ supercell, found

in diffraction experiments, that strengthen the formation of this superstructure in the synthesized crystals.[8] To understand the preferential doping site of Co atoms, we calculated the formation energy of Co in each individual sublattice and list them in Table S1. At the first step, we substituted one Co atom in various sublattices of the √3×√3R30° supercell (6.7% doping concentration). It was found that Fe split sites are the most favored doping sites with -0.72 and -0.61 eV/atom formation energies for FeU and FeD, respectively, where the negative number indicates the spontaneous substitution by Co. The lower formation energy in FeU suggests that Co prefers to decrease the concentration of Fe atoms in the Fe rich side. This is compatible with the previous report on the formation energy of vacancies in FGT,[7] indicating that Co prefers to occupy the same site that is most prone to form vacancy. In the next step, having the Co doped in the FeU site with lowest formation energy, we substitute the second Co atom in the remaining Fe sublattices of this structure (13.4% doping concentration). Once more, substitution in the FeU site was found to be most favored, with a formation energy of -0.67 eV/atom, revealing that Co atoms prefer to sit close to each other if concentration of Fe atoms is equal in both split sites. Here, again the FeD comes as the second best choice, with a formation energy of -0.62 eV/atom. Finally, third Co was added to the 13.4% doped structure to achieve 20.1% doping concentration. However, in this case, all FeU sites are already occupied resulting to additional Co preferentially occupying the FeD split site, with a formation energy of -0.64 eV/atom. Thus, we can assume that up to 20.1%, Co dopant prefers to occupy only the outer most Fe sublattice (split sites) in FGT structure. It can be assumed that experimental samples with 15% Co concentration, should have properties somewhere in the range of simulated 13.4 and 20.1% concentrations. A schematic illustration of these atomic structures is demonstrated in Fig. 1a.

Table S1. *Formation energy of Co substitution in various Fe sublattices as a function of doping concentration, where 1st, 2nd, and 3rd Co correspond to 6.7, 13.4, 20.1% Co concentration.*

**Site**	**Formation Energy (eV/atom)**		
	**1st Co atom**	**2nd Co atom**	**3rd Co atom**
Fe split site up (Fe rich)	**-0.72**	**-0.67**	-
Fe split site down (Fe deficient)	-0.61	-0.62	**-0.64**
Fe2	-0.29	-0.20	-0.17
Fe3	-0.48	-0.47	-0.46
Fe4	-0.52	-0.52	-0.55
Fe5	-0.30	-0.30	-0.31

Table S2 shows that in the absence of Co, pure FGT has an out of plane easy axis, with MAE = +18.7 and +26.1 μeV/atom for monolayer and bulk, respectively. In contrast, as Co gets doped into the structure, the direction of the easy axis changes to in plane for both monolayer and bulk. In the monolayer regime, the MAE values for concentrations of 6.7, 13.4 and 20.1% are -29.6, -79.1 (-93.7) and -149.2 μeV/atom, respectively. One can note the direct relation between the Co concentration and the magnitude of MAE. The same trend can be seen in the bulk with -18.2, -42.3 (-29.3) and -99.8 μeV/atom, for 6.7, 13.4 and 20.1% Co concentration, respectively. Moreover, it can be noted that the vdW proximity effect tends to increase the MAE value in bulk form compared to the monolayer. The value in the parenthesis is the MAE for the second-best energetic structure with 13.4% Co (one FeU and one FeD is doped), indicating that the in plane magnetizaiton is not unique to some special arrangement of Co atoms and the magnetic anisotropy

behaves similarly as far as concentration of Co is kept constant. Our experimental samples are thin films, so we expect that they should behave somewhere in between monolayer and bulk regime, and the consistency between the behavior of monolayer and bulk can be expanded to the thin film regimes.

The atom projected MAE for intermediate concentrations can be found in Supplementary Fig. S1, where the competing interplay between the direction of magnetic anisotropy of various Fe sublattices is persistent. This can also explain the origin of inconsistency between the reported easy axis direction by different experimental groups for various synthesis method and vacancy concentration.[8,9] It is well stablished experimentally, that the Fe split sublattice can be distributed unevenly in up and down sides of each monolayer, depending on the synthesis and quenching condition.[8] Moreover, Fe split sites are the most prone to the formation of vacancies.[7] It can be inferred that high concentration of vacancies can diminish the out of plane contribution of Fe split sites and give rise to in plane magnetization. It was proven both by theory and experiment that Fe_4GeTe_2 which corresponds to Fe_5GeTe_2 with 100% vacancy in Fe split sites has an in plane easy axis.[10,11]

Table S2. *Magnetic anisotropy energy of monolayer and bulk CFGT as a function of concentration of Co, where positive and negative numbers correspond to the out of plane and in plane easy axis respectively. The values in parenthesis are calculated for the second energetically favored structure with 13.4 % Co concentration (corresponding to the occupation of one FeU and one FeD).*

Co percentage	MAE of monolayer (μeV/atom)	MAE of bulk (μeV/atom)
0	+18.7	+26.1
6.7	-29.6	-18.2
13.4	-79.1 (-93.7)	-42.3 (-29.3)
20.1	-149.2	-99.8

Table S3. *Site dependent magnetic moments and atom projected spin polarization for pristine FGT and 20.1 % Co doped CFGT.*

	Pristine Structure		20.1 % Co doped Structure	
Site	Magnetic moment (μB)	Atom projected spin polarization	Magnetic moment (μB)	Atom projected spin polarization
CoU	_	_	0.58	17.5
CoD	_	_	0.80	-9.8
FeU (split Up)	1.53	-31.6	_	_
Fe2	2.55	-16.8	2.64	5.5

Fe3	2.10	-50.0	1.99	-35.2
Fe4	1.97	-28.3	1.87	-34.8
Fe5	2.55	-12.2	2.70	-10.1
FeD (split Down)	1.93	-8.4	_	_

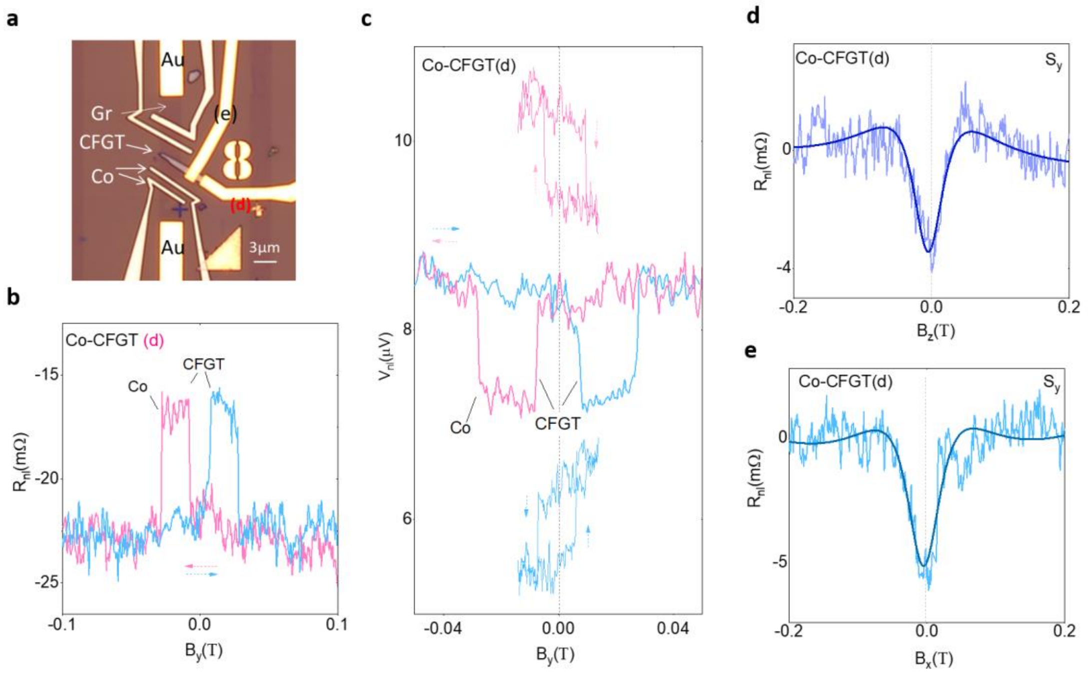

Supplementary Fig. S2. Nonlocal spin-valve measurements in Device 1 using contact d on CFGT at room temperature. a. *Optical image of the lateral spin-valve device, where CFGT (using contact d) acts as a spin detector. The results in the main text are measured using contact e on CFGT.* ***b.*** *Nonlocal spin valve signals with CFGT as the detector measured at room temperature with a bias current of 200 μA. The magnetization direction switchings of CFGT and Co are indicated. The blue and pink data are for up- and down- sweeps of B_y, as indicated by the dashed arrows.* ***c.*** *Minor-loop measurement showing memory effect of CFGT with B_y field sweep for forward and backward directions with a comparison with the full spin valve signal at a bias curent of -200 μA.* ***d,e.*** *The measured z- and x-Hanle spin precession signals show the dominant S_y spin polarization component. Linear background was subtracted from the data. The smooth darker line is the standard Hanle fitting result. All the measurements were performed in Dev 1 at room temperature.*

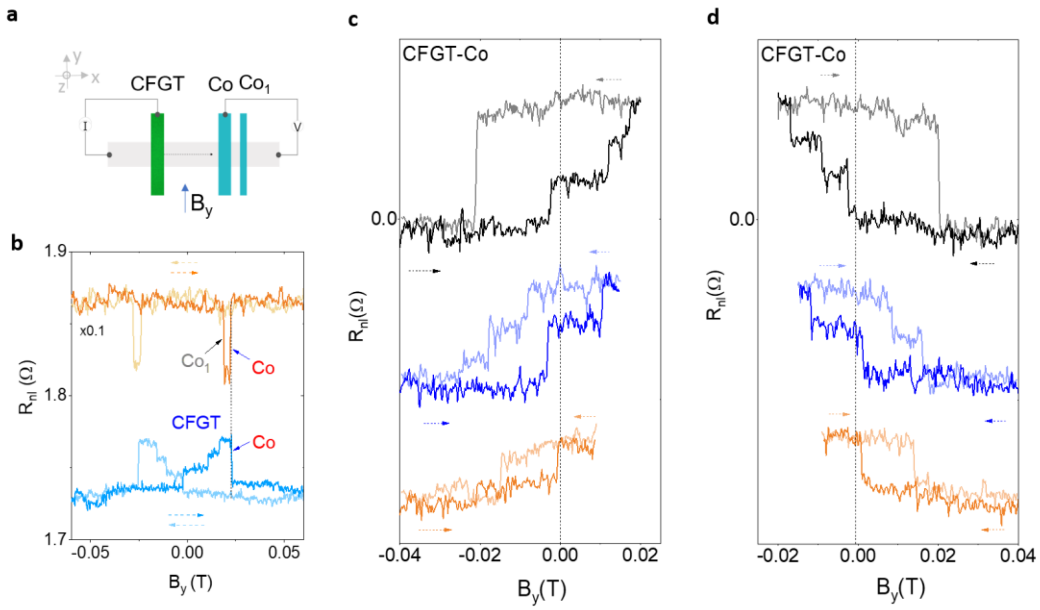

Supplementary Fig. S3. Spin-valve signal with CFGT as spin injector using Device 2 at room temperature. a. *Device schematics using CFGT as injector and Co as detector in a nonlocal spin valve measurement set-up.* ***b.*** *Comparison between the lateral nonlocal CFGT-Co and Co_1-Co device spin valve signals. The Co_1-Co signal was multiplied by 0.1 for clarity. From the two spin valve signals, the sharp switch at higher field is identified to be from Co while the multiple switches at low fields are attributed to different magnetic domains in the CFGT.* ***c,d.*** *Minor loop for up-sweep and down-sweep showing the memory property of Dev 2. The step-like switching observed is consistent with the full spin valve signal in (b). Measurements are performed in Dev 2 at room temperature.*

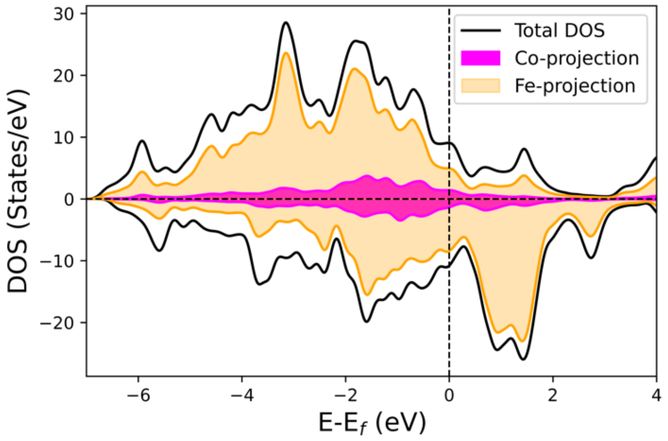

Supplementary Fig. S4. *Density of states (DOS) – solid black line and projected density of states (pDOS) – colored shaded, for 13.4% doped CFGT, where orange represents the density of Fe states and violet represents the density of Co states.*

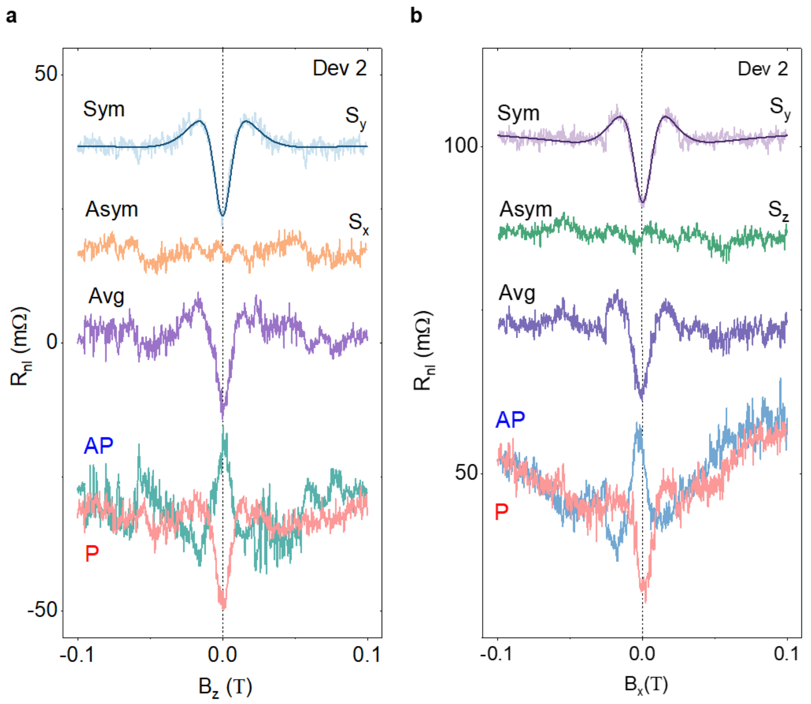

Supplementary Fig. S5. Detailed z-Hanle and x-Hanle measurements for Dev 2. *a.* *z-Hanle spin precession signal for Dev 2, using CFGT as an injector. z-Hanle signals were measured in the CFGT-Co spin valve with parallel (P) and antiparallel (AP) magnetic configurations of electrodes. The averaged (Avg) Hanle signal and extracted symmetric (Sym) and anti-symmetric (Asym) components, corresponding to S_y and S_x, respectively, showing clear symmetric signal.* ***b*** *x-Hanle measurement performed for Dev 2, with the raw data obtained using parallel and antiparallel configurations of injector and detector. Similarly, the Avg Hanle signal and Sym and Asym components, corresponding to S_y and S_z, respectively, are presented. A linear background is subtracted from the measured data. All measurements are performed at room temperature.*

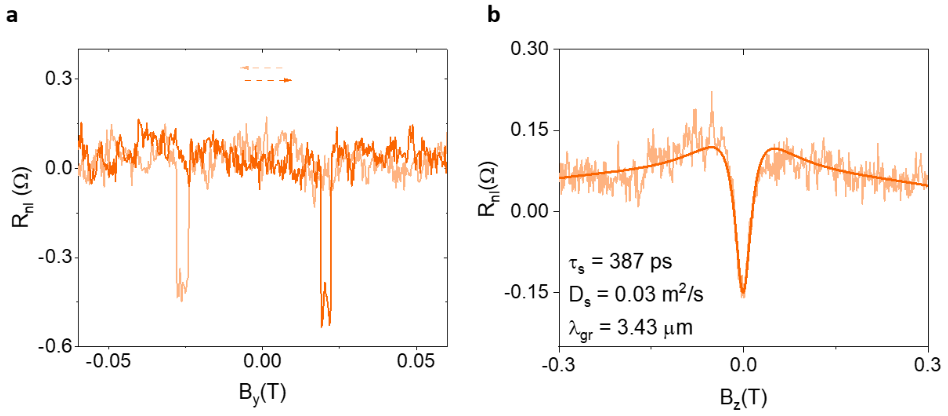

Supplementary Fig. S6. *Spin transport in Co-Co (injector-detector) reference for Dev 2.* *a) Spin valve signal wih up-sweep and down-sweep of applied magnetic field B_y indicated by arrows. b) z-Hanle spin precession signal (light orange) with Hanle fitting curve (dark orange). Spin lifetime τ_s, spin diffusion constant D_s, and spin diffusion length λ_{gr} in the graphene channel was extracted from the fitting curve.*

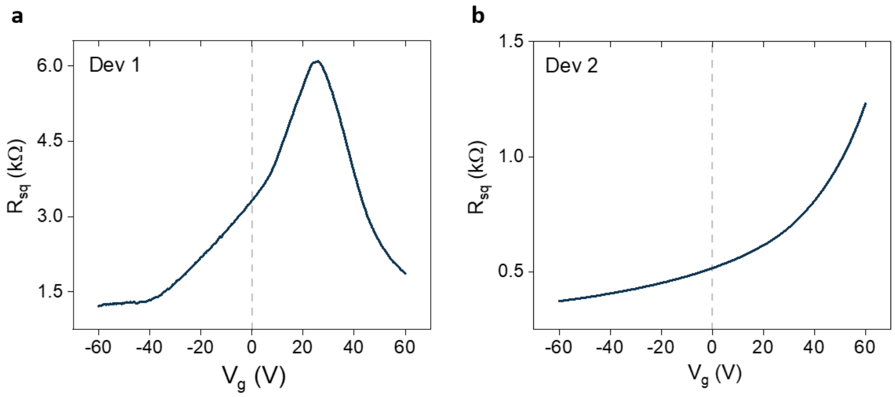

Supplementary Fig. S7. *Square resistance in the graphene channel for a) Dev 1 and b) Dev 2. All spin transport measurements where performed at* $V_g = 0\,V$*, as marked by the dashed lines.*

References

(1) Kresse, G.; Hafner, J. Norm-Conserving and Ultrasoft Pseudopotentials for First-Row and Transition Elements. *J. Phys. Condens. Matter* **1994**, 6(40)

(2) Kresse, G.; Joubert, D. From Ultrasoft Pseudopotentials to the Projector Augmented-Wave Method. *Phys Rev B* **1999**, *59* (3), 1758–1775.

(3) Perdew, J. P.; Burke, K.; Ernzerhof, M. Generalized Gradient Approximation Made Simple. *Phys. Rev. Lett.* **1996**, *77*, 3865–3868.

(4) Monkhorst, H. J.; Pack, J. D. Special Points for Brillonin-Zone Integrations*. *Phys. Rev. B* **1976**, *13* (12), 5188–5192.

(5) Smidstrup, S.; Markussen, T.; Vancraeyveld, P.; Wellendorff, J.; Schneider, J.; Gunst, T.; Verstichel, B.; Stradi, D.; Khomyakov, P. A.; Vej-Hansen, U. G.; Lee, M. E.; Chill, S. T.; Rasmussen, F.; Penazzi, G.; Corsetti, F.; Ojanperä, A.; Jensen, K.; Palsgaard, M. L. N.; Martinez, U.; Blom, A.; Brandbyge, M.; Stokbro, K. QuantumATK: An Integrated Platform of Electronic and Atomic-Scale Modelling Tools. *Journal of Physics Condensed Matter* **2020**, *32* (1).

(6) van Setten, M. J.; Giantomassi, M.; Bousquet, E.; Verstraete, M. J.; Hamann, D. R.; Gonze, X.; Rignanese, G. M. The PSEUDODOJO: Training and Grading a 85 Element Optimized Norm-Conserving Pseudopotential Table. *Comput Phys Commun* **2018**, *226*, 39–54.

(7) Ershadrad, S.; Ghosh, S.; Wang, D.; Kvashnin, Y.; Sanyal, B. Unusual Magnetic Features in Two-Dimensional Fe5GeTe2Induced by Structural Reconstructions. *Journal of Physical Chemistry Letters* **2022**, *13*, 4877–4883.

(8) May, A. F.; Ovchinnikov, D.; Zheng, Q.; Hermann, R.; Calder, S.; Huang, B.; Fei, Z.; Liu, Y.; Xu, X.; McGuire, M. A. Ferromagnetism Near Room Temperature in the Cleavable van Der Waals Crystal Fe 5 GeTe 2. *ACS Nano* **2019**, *13* (4), 4436–4442.

(9) Zhang, H.; Chen, R.; Zhai, K.; Chen, X.; Caretta, L.; Huang, X.; Chopdekar, R. V.; Cao, J.; Sun, J.; Yao, J.; Birgeneau, R.; Ramesh, R. Itinerant Ferromagnetism in van Der Waals Fe5-XGeTe2 Crystals above Room Temperature. *Phys Rev B* **2020**, *102* (6), 64417.

(10) Ghosh, S.; Ershadrad, S.; Borisov, V.; Sanyal, B. Unraveling Effects of Electron Correlation in Two-Dimensional FenGeTe2 (N=3, 4, 5) by Dynamical Mean Field Theory. *npj Comput Mater* **2023**, *9* (86), 1–16.

(11) Seo, J.; Kim, D. W. D. Y.; An, E. S.; Kim, K.; Kim, G. Y.; Hwang, S. Y.; Kim, D. W. D. Y.; Jang, B. G.; Kim, H.; Eom, G.; Seo, S. Y.; Stania, R.; Muntwiler, M.; Lee, J. J.; Watanabe, K.; Taniguchi, T.; Jo, Y. J.; Lee, J. J.; Min, B. Il; Jo, M. H.; Yeom, H. W.; Choi, S. Y.; Shim, J. H.; Kim, J. S. Nearly

Room Temperature Ferromagnetism in a Magnetic Metal-Rich van Der Waals Metal. *Sci Adv* **2020**, *6* (3), 1–10.
